# Pop‐off mechanisms in fetal megacystis: extravasation, umbilical cord cyst, ureterocele and megaureter

**DOI:** 10.1002/uog.29200

**Published:** 2025-03-04

**Authors:** L. A. M. Brinkman, L. K. Duin, P. N. Adama van Scheltema, T. E. Cohen‐Overbeek, E. Pajkrt, M. N. Bekker, C. Willekes, E. J. T. Verweij, C. Bilardo, F. Fontanella

**Affiliations:** ^1^ Department of Obstetrics and Gynecology, University Medical Center Groningen University of Groningen Groningen The Netherlands; ^2^ Department of Obstetrics, Gynecology and Prenatal Diagnosis Leiden University Medical Center Leiden The Netherlands; ^3^ Department of Obstetrics and Gynecology, Division of Obstetrics and Fetal Medicine Erasmus MC‐Sophia Children's Hospital University Medical Center Rotterdam The Netherlands; ^4^ Department of Obstetrics and Gynecology, Amsterdam University Medical Center University of Amsterdam Amsterdam The Netherlands; ^5^ Department of Obstetrics, Gynecology and Prenatal Diagnosis University Medical Center Utrecht Utrecht The Netherlands; ^6^ Department of Obstetrics, Gynecology and Prenatal Diagnosis University Medical Center, Grow School for Oncology and Medical Biology Maastricht The Netherlands

**Keywords:** antenatal diagnosis, lower urinary tract obstruction, LUTO, renal function, ultrasound

## Abstract

**Objective:**

To analyze comprehensively the incidence, antenatal ultrasound characteristics and prognostic implications of antenatal pop‐off mechanisms of the fetal urinary system in pregnancies with suspected fetal megacystis.

**Methods:**

This was a retrospective multicenter study of pregnancies with suspected fetal megacystis conducted across all academic hospitals in The Netherlands. Three antenatal pop‐off mechanisms were identified: presence of an umbilical cord cyst (UCC), extravasation of urine into the intraperitoneal space (ascites) or perirenal subcapsular (urinoma), and megaureter/ureterocele. Cases that exhibited two different pop‐off mechanisms, underwent vesicoamniotic shunt placement or had unclear information regarding shunt placement were excluded. We compared the antenatal ultrasound characteristics and outcomes among pregnancies with UCC, those with extravasation, those with megaureter/ureterocele and those without a pop‐off mechanism. Logistic regression analysis was used to evaluate the association of pop‐off mechanisms with antenatal characteristics and postnatal outcomes.

**Results:**

Among 543 fetuses with suspected megacystis, 76% exhibited no pop‐off mechanism, 7% presented with UCC only, 9% presented with extravasation only, 7% presented with a megaureter/ureterocele only and 1% presented with two pop‐off mechanisms. Following exclusions, 511 cases were included in the analysis. The identification of UCC (*n* = 39) was associated with early‐onset megacystis (odds ratio (OR), 4.2 (95% CI, 1.9–9.1); *P* < 0.001), severe megacystis (OR 2.3 (95% CI, 1.1–5.0); *P* = 0.033), normal amniotic fluid index (AFI) (OR, 3.3 (95% CI, 1.3–8.2); *P* = 0.011) and additional associated anomaly (OR, 3.3 (95% CI, 1.7–6.4); *P* < 0.001), and thus with the highest prevalence of complex diagnosis (66%), primarily represented by anorectal malformation. Extravasation (*n* = 42) was associated with severe megacystis (OR, 2.4 (95% CI, 1.1–5.4); *P* = 0.030), abnormal AFI (OR, 2.8 (95% CI, 1.2–6.8); *P* = 0.022), the keyhole sign (OR, 2.5 (95% CI, 1.1–5.8); *P* = 0.033) and additional associated anomaly (OR, 2.1 (95% CI, 1.1–4.1); *P* = 0.026). Megaureter/ureterocele (*n* = 36) was associated with late‐onset megacystis (OR, 4.0 (95% CI, 1.6–9.7); *P* = 0.003), a thickened bladder wall during pregnancy (OR, 6.6 (95% CI, 1.9–23.1); *P* = 0.003) and the lowest prevalence of additional associated anomaly (22%).

Intrauterine fetal demise was most prevalent in fetuses with UCC (15%), while termination of pregnancy and non‐survivors were most common in cases with extravasation (50% and 17%, respectively). The majority of fetuses with megacystis associated with megaureter/ureterocele were still alive during follow‐up (72%) and the odds of survival were the highest for this group (OR, 2.7 (95% CI, 1.3–5.7); *P* = 0.010).

**Conclusions:**

Antenatal pop‐off mechanisms may alleviate high intraluminal pressure within the fetal urinary tract. Each mechanism leads to a different antenatal clinical picture and outcome, which may explain partially the heterogeneity of outcomes in fetuses with megacystis. Therefore, understanding the implications of these mechanisms and their antenatal characteristics could guide antenatal counseling and management of fetal megacystis. © 2025 The Author(s). *Ultrasound in Obstetrics & Gynecology* published by John Wiley & Sons Ltd on behalf of International Society of Ultrasound in Obstetrics and Gynecology.

## INTRODUCTION

Fetal megacystis, identifiable as early as 10 weeks' gestation, is characterized by the presence of a dilated fetal urinary bladder and may result from lower urinary tract obstruction (LUTO), a chromosomal anomaly or another congenital anomaly^1^, ^2^, ^3^. Although pregnancies with severe megacystis are often terminated, milder degrees of bladder dilatation may resolve spontaneously during pregnancy with favorable postnatal outcome^1^. In severe cases, fetal megacystis has been associated with renal failure, oligohydramnios and pulmonary hypoplasia^2^. Due to its diverse etiology and unpredictable evolution, the management and counseling of fetal megacystis remain challenging^1^, ^2^.

With the natural progression of fetal megacystis, a spectrum of ‘pop‐off’ mechanisms has been observed, reflecting an attempt to alleviate elevated intraluminal urinary pressure and thus protect the renal parenchyma. These mechanisms include the development of an umbilical cord cyst (UCC), which is sometimes detectable sonographically even before the bladder can be identified at 10 weeks' gestation^4^. Additionally, urine may extravasate into the intraperitoneal space (ascites) or the perirenal subcapsular area (perinephritic urinoma) due to rupture of the renal or upper urinary tract, often resulting in irreversible renal damage. Another pop‐off mechanism is the formation of a megaureter or ureterocele.

These natural pop‐off mechanisms may align with the rationale behind antenatal intervention, which aims to reduce intraluminal pressure and thereby mitigate renal damage. Moreover, these mechanisms may contribute to the vast variability in the antenatal course of fetal megacystis and its postnatal outcome.

Previous studies have focused mainly on the postnatal incidence of pop‐off mechanisms in infants with LUTO^5^. However, despite their potential relevance in protecting the renal parenchyma during fetal life, the incidence, typical ultrasound appearance and outcomes of pop‐off mechanisms during pregnancy remain largely unexplored. Consequently, their specific roles in determining perinatal and long‐term outcomes remain unclear. It is uncertain whether pop‐off mechanisms offer protection against harmful effects on the urinary system or signify a severe insult which leads to urinary and bladder dysfunction later in life. This study aimed to provide a thorough evaluation of the incidence, antenatal ultrasound characteristics and outcomes of pop‐off mechanisms identified in fetuses with suspected megacystis.

## METHODS

### Study design and data collection

This retrospective multicenter study was carried out at the fetal medicine units (FMUs) of all academic hospitals in The Netherlands. Data on cases of suspected fetal megacystis were extracted from local databases from the point of digital registration inception in each respective database: from 2000 to the end of 2014 in three centers (Academic Medical Center Amsterdam, Amsterdam; Erasmus MC‐Sophia Children's Hospital University Medical Center, Rotterdam; University Medical Center Maastricht, Maastricht), from 2004 to the end of 2014 in two centers (Radboud University Medical Center, Nijmegen; University Medical Center Groningen, Groningen) and from 2007 to the end of 2014 in three centers (Utrecht University Medical Center, Utrecht; Leiden University Medical Center, Leiden; VU University Medical Center, Amsterdam). This study was approved by the medical ethics committee of the University Medical Center Groningen (METc 2015/445).

In The Netherlands, when there is suspicion of fetal megacystis, referral to a FMU is indicated for confirmation of diagnosis and further investigation. These referrals typically occur following a dating scan, first‐trimester scan, 20‐week anomaly scan or a scan performed later in gestation for another indication. Each referred case underwent a detailed anomaly scan, and parents were counseled on the prognosis of the condition. Information about potential *in‐utero* treatment options was offered to parents with a male fetus with normal chromosomal analysis, displaying isolated signs of LUTO and with concomitant oligohydramnios. Fetuses that underwent vesicoamniotic shunt placement or cases for which reported information regarding shunt placement was unclear were excluded from data analysis. Pre‐ and postnatal clinical data were collected from the medical records of both the mother and child.

### Definitions

In this study, we considered three antenatal pop‐off mechanisms. First, UCC was defined broadly as a hypoechogenic area within the umbilical cord or (suspicion of) a patent urachus, accompanied by the presence of an allantoic cyst. Second, extravasation was defined as ascites (caused by rupture or leakage of the distended bladder) and/or the presence of a urinoma, meaning extravasation of urine into the intraperitoneal space or perirenal subcapsular, respectively, resulting from high intraluminal pressure^6^, ^7^. Third, a ureterocele or megaureter was defined as the (intravesical cystic) dilatation of the ureter or dilatation of the portion of the ureter between the renal pelvis and bladder insertion. In the case of a ureterocele, the sonographic presentation may also include a duplex kidney, which indicates duplication of the renal collecting system. This additional finding can be an important diagnostic feature when evaluating the extent and implications of the ureterocele.

Measurements of the longitudinal bladder diameter (LBD) were conducted in a midsagittal view, by measuring the distance from the fetal bladder dome to the bladder neck. Fetal megacystis was defined as a LBD ≥ 7 mm in the first trimester^8^. After 14 weeks' gestation, a bladder was considered dilated if it failed to empty over a period of at least 45 min^8^, ^9^. Furthermore, our study distinguished between early‐onset megacystis, defined as a diagnosis before 18 weeks' gestation, and late‐onset megacystis, defined as a diagnosis at or after 18 weeks' gestation. Moreover, severe megacystis was defined as a LBD ≥ 14 mm before 15 weeks' gestation or as a *Z*‐score > 5.2 at or after 15 weeks up to and including week 35 of gestation, according to published reference charts^10^. The keyhole sign was considered to represent a dilatated posterior urethra. The bladder wall was considered ‘thickened’ if > 2 mm, or if labeled as such by the fetal medicine specialist in charge. The uterus was divided into four quadrants to measure the amniotic fluid index (AFI). An AFI < 5 cm was defined as abnormal^11^.

All postnatal investigations and postmortem examinations were reviewed thoroughly to establish a final diagnosis. LUTO was defined as a bladder outlet obstruction caused by urethral stenosis, urethral valves or urethral atresia. Cases with LUTO reported as the final diagnosis, but without further details concerning the type of obstruction, were defined as non‐specified LUTO. Regarding the etiology of the megacystis, infants with neither LUTO nor a congenital syndrome/severe congenital anomaly were categorized as having a normal urinary tract. The group with complex megacystis included fetuses with trisomy 13, 18 or 21, anorectal malformation (ARM), complex LUTO or another congenital syndrome/chromosomal anomaly (for example, Turner syndrome or overgrowth syndrome). ARM describes a group of complex congenital anomalies marked by abnormal development of the urorectal septum, resulting in congenital abnormalities of the genitourinary tract, rectum and distal anus, such as cloacal malformation and VACTERL association^12^.

Associated structural anomalies were divided into minor and severe anomalies. Minor associated anomalies included single umbilical artery, echogenic bowel, choroid plexus cyst, macroglossia, hypoplastic nasal bone, shortened long bones, increased nuchal translucency thickness, isolated ventriculomegaly, strawberry sign and arachnoid cyst. Severe associated anomalies included cardiac anomaly, hydrops, skeletal anomaly, renal agenesis, genital anomaly, hygroma colli, abdominal wall defect, central nervous system anomaly, diaphragmatic hernia and sacrococcygeal teratoma.

Termination of pregnancy was defined as the choice by parents to end the pregnancy by medical intervention or induction before 24 weeks' gestation. Intrauterine fetal demise (IUFD) was defined as fetal death after 20 weeks' gestation. Non‐survivor was defined as the death of a liveborn infant during follow‐up.

Estimated glomerular filtration rate (eGFR), as a measure of postnatal renal function, was calculated using the Schwartz formula, considering the length of the infant and the creatinine nadir within the first year of diagnosis, as the literature has shown this to be a good long‐term predictor for renal function^13^, ^14^. eGFR ≥ 90 mL/min/1.73 m^2^ was classified as normal and eGFR < 90 mL/min/1.73 m^2^ was classified as abnormal.

### Statistical analysis

Data are presented as median (interquartile range (IQR)) or *n* (%). The Kruskal–Wallis test was used to compare continuous variables. The chi‐square test or Fisher's exact test was used to compare categorical variables. A *P*‐value ≤ 0.05 was considered statistically significant. Logistic regression analysis was used to evaluate the association of pop‐off mechanisms with antenatal characteristics and postnatal outcomes, and results are expressed as odds ratios (ORs) with 95% CI. Statistical Package for the Social Sciences (SPSS) version 28.0 (IBM Corp., Armonk, NY, USA) was employed for statistical analysis.

## RESULTS

### Patient characteristics

**Figure 1:**
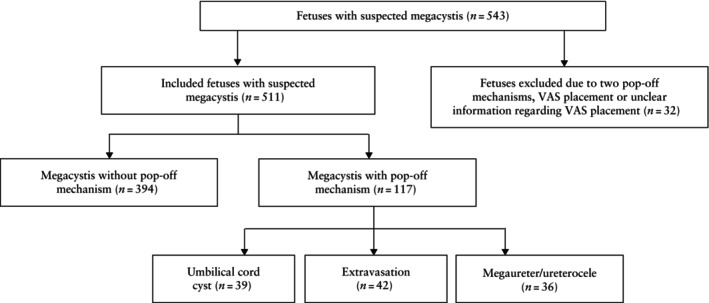
Flowchart summarizing inclusion of cases with suspected fetal megacystis in the study population. VAS, vesicoamniotic shunt.

In total, 543 fetuses with suspicion of megacystis were identified. Among these cases, the majority (74%) were male and the median gestational age at diagnosis was 20.0 (IQR, 13.3–25.6) weeks (Table 1). Within this cohort, 413 (76%) fetuses exhibited no pop‐off mechanism, 39 (7%) presented with UCC only, 47 (9%) presented with extravasation only, 39 (7%) presented with a megaureter/ureterocele only and 5 (1%) fetuses exhibited two pop‐off mechanisms. This latter group (*n* = 5) was excluded from analysis, as were 27 fetuses who received vesicoamniotic shunt placement or had unclear information reported regarding shunt placement, leaving 511 fetuses for analysis (Figure 1). Further details concerning the underlying etiology and outcome of the study population have been published previously^1^.

**Table 1:** Demographic and clinical characteristics of 543 pregnancies with suspicion of fetal megacystis

Characteristic	Value
Gestational age at diagnosis* (weeks)	20.00 (13.29–25.61)
Pop‐off mechanism	
None	413 (76.1)
Umbilical cord cyst only	39 (7.2)
Extravasation only	47 (8.7)
Megaureter/ureterocele only	39 (7.2)
Two mechanisms	5 (1.0)
eGFR (mL/min/1.73 m^2^)†, ‡	85.50 (47.87–109.53)

Data are given as median (interquartile range) or *n* (%).

* Data missing for 5 patients (*n* = 538).

† Data missing for 416 patients (*n* = 127).

‡ Estimated glomerular filtration rate (eGFR) was based on the Schwartz formula.

### Umbilical cord cyst

Among the 39/511 (8%) fetuses with UCC, diagnosis occurred at a median gestational age of 13.6 (IQR, 12.1–17.0) weeks. Compared to the group with no pop‐off mechanism, fetuses with UCC had a significantly higher prevalence (77% *vs* 44%) and higher odds (OR, 4.2 (95% CI, 1.9–9.1); *P* < 0.001) of early‐onset megacystis (Tables 2 and 3). Furthermore, UCC was associated with severe megacystis (OR, 2.3 (95% CI, 1.1–5.0); *P* = 0.033). Fetuses with megacystis associated with UCC were more often characterized by a normal AFI (72%; OR, 3.3 (95% CI, 1.3–8.2); *P* = 0.011) and had the lowest prevalence of a thickened bladder wall (29%) compared to the groups with other pop‐off mechanisms.

**Table 2:** Antenatal characteristics and postnatal outcomes in 511 pregnancies with suspected fetal megacystis, according to presence and type of pop‐off mechanism

Characteristic	No pop‐off mechanism (*n* = 394)	Umbilical cord cyst (*n* = 39)	Extravasation (*n* = 42)	Megaureter/ureterocele (*n* = 36)	*P*
GA at diagnosis* (weeks)	19.86 (13.14–26.57)	13.57 (12.14–17.00)	20.50 (16.53–23.04)	21.22 (20.04–33.15)	< 0.001
Onset of megacystis					< 0.001
Early (< 18 weeks)	173/391 (44.2)	30/39 (76.9)	13/42 (31.0)	6/36 (16.7)	
Late (≥ 18 weeks)	218/391 (55.8)	9/39 (23.1)	29/42 (69.0)	30/36 (83.3)	
LBD (mm)†	30.00 (18.00–45.00)	25.05 (16.00–34.75)	40.00 (26.23–56.00)	29.50 (23.30–46.00)	0.026
Severity of megacystis‡					0.030
Non‐severe	151/276 (54.7)	11/32 (34.4)	10/30 (33.3)	11/20 (55.0)	
Severe	125/276 (45.3)	21/32 (65.6)	20/30 (66.7)	9/20 (45.0)	
Keyhole sign present	130/238 (54.6)	9/21 (42.9)	24/32 (75.0)	16/24 (66.7)	0.060
Thickened bladder wall	89/192 (46.4)	6/21 (28.6)	14/25 (56.0)	17/20 (85.0)	0.002
Amniotic fluid index					0.002
Normal (≥ 5 cm)	95/216 (44.0)	18/25 (72.0)	7/32 (21.9)	9/16 (56.3)	
Abnormal (< 5 cm)	121/216 (56.0)	7/25 (28.0)	25/32 (78.1)	7/16 (43.8)	
Megacystis					0.029
Isolated	212/325 (65.2)	14/33 (42.4)	21/38 (55.3)	24/33 (72.7)	
Non‐isolated	113/325 (34.8)	19/33 (57.6)	17/38 (44.7)	9/33 (27.3)	
Etiology of megacystis§					< 0.001
Normal urinary tract	39/289 (13.5)	2/32 (6.3)	1/36 (2.8)	15/33 (45.5)	
Isolated LUTO	155/289 (53.6)	9/32 (28.1)	21/36 (58.3)	15/33 (45.5)	
Complex diagnosis	95/289 (32.9)	21/32 (65.6)	14/36 (38.9)	3/33 (9.1)	
Trisomy	26	2	3	0	
Anorectal malformation	14	12	4	0	
Cause of obstruction in LUTO					0.025
Non‐specified LUTO	87/162 (53.7)	5/14 (35.7)	14/23 (60.9)	7/15 (46.7)	
PUV	63/162 (38.9)	5/14 (35.7)	7/23 (30.4)	7/15 (46.7)	
Urethral stenosis	9/162 (5.6)	0/14 (0)	0/23 (0)	1/15 (6.7)	
Urethral atresia	3/162 (1.9)	4/14 (28.6)	2/23 (8.7)	0/15 (0)	
Additional associated anomaly	96/394 (24.4)	20/39 (51.3)	17/42 (40.5)	8/36 (22.2)	< 0.001
Minor	38/96 (39.6)	9/20 (45.0)	5/17 (29.4)	4/8 (50.0)	
Severe	58/96 (60.4)	11/20 (55.0)	12/17 (70.6)	4/8 (50.0)	
Outcome					< 0.001
Termination of pregnancy	143/385 (37.1)	17/39 (43.6)	21/42 (50.0)	7/36 (19.4)	
Intrauterine fetal demise	27/385 (7.0)	6/39 (15.4)	5/42 (11.9)	1/36 (2.8)	
Non‐survivor	26/385 (6.8)	3/39 (7.7)	7/42 (16.7)	2/36 (5.6)	
Alive during follow‐up	189/385 (49.1)	13/39 (33.3)	9/42 (21.4)	26/36 (72.2)	
eGFR					0.546
Normal (≥ 90 mL/min/1.73 m^2^)	41/88 (46.6)	5/9 (55.6)	3/6 (50.0)	10/15 (66.7)	
Abnormal (< 90 mL/min/1.73 m^2^)	47/88 (53.4)	4/9 (44.4)	3/6 (50.0)	5/15 (33.3)	

Data are given as median (interquartile range), *n*/N (%) or *n*.

* Data missing for three patients with no pop‐off mechanism.

† Data missing for 111 patients with no pop‐off mechanism, 7 patients with umbilical cord cyst, 12 patients with extravasation and 15 patients with megaureter/ureterocele.

‡ Severe megacystis defined as longitudinal bladder diameter (LBD) ≥ 14 mm before 15 weeks' gestation or *Z*‐score > 5.2 at or after 15 weeks up to and including week 35 of gestation.

§ Established on postnatal investigation or postmortem examination.

eGFR, estimated glomerular filtration rate; GA, gestational age; LUTO, lower urinary tract obstruction; PUV, posterior urethral valves.

**Table 3:** Logistic regression analysis of association of antenatal characteristics and postnatal outcomes with different pop‐off mechanisms in 511 pregnancies with suspected fetal megacystis

	Umblical cord cyst		Extravasation		Megaureter/ureterocele	
Characteristic	OR (95% CI)	*P*	OR (95% CI)	*P*	OR (95% CI)	*P*
Early‐onset megacystis	4.200 (1.943–9.082)	< 0.001	0.565 (0.285–1.119)	0.102	0.252 (0.103–0.619)	0.003
Severe megacystis*	2.306 (1.071–4.966)	0.033	2.416 (1.091–5.351)	0.030	0.988 (0.397–2.461)	0.980
Keyhole sign present	0.623 (0.253–1.534)	0.304	2.492 (1.076–5.772)	0.033	1.662 (0.685–4.031)	0.261
Thickened bladder wall	0.463 (0.172–1.244)	0.127	1.473 (0.636–3.409)	0.366	6.558 (1.861–23.114)	0.003
Abnormal AFI†	0.305 (0.122–0.761)	0.011	2.804 (1.163–6.761)	0.022	0.611 (0.219–1.700)	0.345
Isolated megacystis	0.393 (0.190–0.813)	0.012	0.658 (0.334–1.298)	0.228	1.421 (0.639–3.161)	0.389
Additional associated anomaly	3.268 (1.674–6.377)	< 0.001	2.111 (1.094–4.075)	0.026	0.887 (0.391–2.011)	0.774
Severe associated anomaly	0.801 (0.303–2.115)	0.654	1.572 (0.513–4.822)	0.429	0.655 (0.154–2.779)	0.566
Alive during follow‐up	0.519 (0.259–1.039)	0.064	0.283 (0.132–0.607)	0.001	2.696 (1.266–5.743)	0.010
Abnormal eGFR‡	0.698 (0.176–2.774)	0.609	0.872 (0.167–4.561)	0.871	0.436 (0.138–1.381)	0.158

Reference category is no pop‐off mechanism.

* Defined as longitudinal bladder diameter ≥ 14 mm before 15 weeks' gestation or *Z*‐score > 5.2 at or after 15 weeks up to and including week 35 of gestation.

† Defined as amniotic fluid index (AFI) < 5 cm.

‡ Defined as estimated glomerular filtration rate (eGFR) < 90 mL/min/1.73 m^2^.

OR, odds ratio.

The etiology of megacystis was established from postnatal investigation or postmortem examination in 32/39 (82%) fetuses, of which 21 (66%) had complex megacystis, 9 (28%) had isolated LUTO and 2 (6%) had a normal urinary tract. ARM was the most common underlying etiology among cases in the UCC group with a complex diagnosis (*n* = 12). Additionally, UCC was associated with the presence of other anomaly (OR, 3.3 (95% CI, 1.7–6.4); *P* < 0.001), and was the sole pop‐off mechanism for which over 50% of cases manifested additional associated anomaly. Among the cases with additional associated anomaly, 45% were minor and 55% were severe. Compared to fetuses with no or another pop‐off mechanism, fetuses with megacystis associated with UCC experienced the highest rate of IUFD (15%).

### Extravasation

Extravasation was noted in 42/511 (8%) fetuses, for which the median gestational at diagnosis was 20.5 (IQR, 16.5–23.0) weeks (Table 2). The majority (69%) of fetuses with extravasation exhibited late‐onset megacystis. The highest frequency (67%) and highest odds (OR, 2.4 (95% CI, 1.1–5.4); *P* = 0.030) for severe megacystis were reported in this group. Furthermore, compared to fetuses with no or another pop‐off mechanism, this group had the highest prevalence (78%) of abnormal AFI and highest odds (OR, 2.8 (95% CI, 1.2–6.8); *P* = 0.022) (Tables 2 and 3). Moreover, fetal megacystis with extravasation was associated with the presence of the keyhole sign (OR, 2.5 (95% CI, 1.1–5.8); *P* = 0.033).

The etiology of megacystis was established from postnatal investigation or postmortem examination in 36/42 (86%) cases, of which the majority (*n* = 21 (58%)) had isolated LUTO. A normal urinary tract was observed in only one case (3%) in this group. A complex diagnosis was identified as the underlying etiology in 14 (39%) cases, including 4 cases of ARM and 3 cases of trisomy. Furthermore, extravasation was significantly associated with other anomaly (OR, 2.1 (95% CI, 1.1–4.1); *P* = 0.026). Among cases with additional associated anomaly, fetuses with extravasation exhibited the highest prevalence of severe associated anomaly (71%) compared to fetuses with no or another pop‐off mechanism. These fetuses also had the highest rates of pregnancy termination (50%) and non‐survivors (17%). Consequently, survival was less likely in this group, with decreased odds of survival (OR, 0.3 (95% CI, 0.1–0.6); *P* = 0.001).

### Megaureter/ureterocele

Megacystis associated with a megaureter/ureterocele was identified in 36/511 (7%) fetuses. Diagnosis occurred at a median gestational age of 21.2 (IQR, 20.0–33.2) weeks, which was notably later compared with the other groups (Table 2). Consequently, megaureter/ureterocele was associated with late‐onset megacystis (OR, 4.0 (95% CI, 1.6–9.7); *P* = 0.003). This group had the highest prevalence of non‐severe megacystis (55%), and the highest prevalence (85%) and highest odds (OR, 6.6 (95% CI, 1.9–23.1); *P* = 0.003) for a thickened bladder wall (Table 3).

The etiology of megacystis was established from postnatal investigation or postmortem examination in 33/36 (92%) of cases. With respect to the etiology of megacystis, this group exhibited the highest frequency of a normal urinary tract (45%) and the lowest frequency of complex diagnosis (9%), meaning that additional associated anomaly was less common in this group (22%). Isolated LUTO was also a common underlying etiology of megacystis in this group (45%), largely caused by posterior urethral valves (PUV) (47%).

Regarding postnatal outcomes, cases with a megaureter/ureterocele had the highest survival rate (72%) compared with other groups and had the highest odds of being alive during follow‐up (OR, 2.7 (95% CI, 1.3–5.7); *P* = 0.010). Finally, the highest rate of normal postnatal eGFR was reported in this group (67%), although this finding was not statistically significant.

## DISCUSSION

This is the first study to investigate the incidence, ultrasound findings and clinical implications of three antenatal pop‐off mechanisms in pregnancies with suspected fetal megacystis. UCC is associated with early‐onset and severe megacystis, normal AFI and additional associated anomaly, and these cases had the highest rate of IUFD. Extravasation was associated with severe megacystis, abnormal AFI, the keyhole sign and additional associated anomaly, and these cases had the highest rates of pregnancy termination and non‐survivors. Megaureter/ureterocele was associated with late‐onset megacystis and a thickened bladder wall, and these cases had the highest postnatal survival rate.

Pop‐off mechanisms, including ascites and urinoma, have been reported previously in approximately 30% of infants with PUV^5^. Our study investigated their occurrence in fetuses with megacystis and found a prevalence of 23%. Antenatal decompression, facilitated either by intervention or a natural pop‐off mechanism, may protect the renal parenchyma from increased intraluminal pressure^15^. It can be hypothesized that fetuses with increased intraluminal pressure that do not exhibit a pop‐off mechanism may experience worse outcomes.

### Umbilical cord cyst

**Figure 2:**
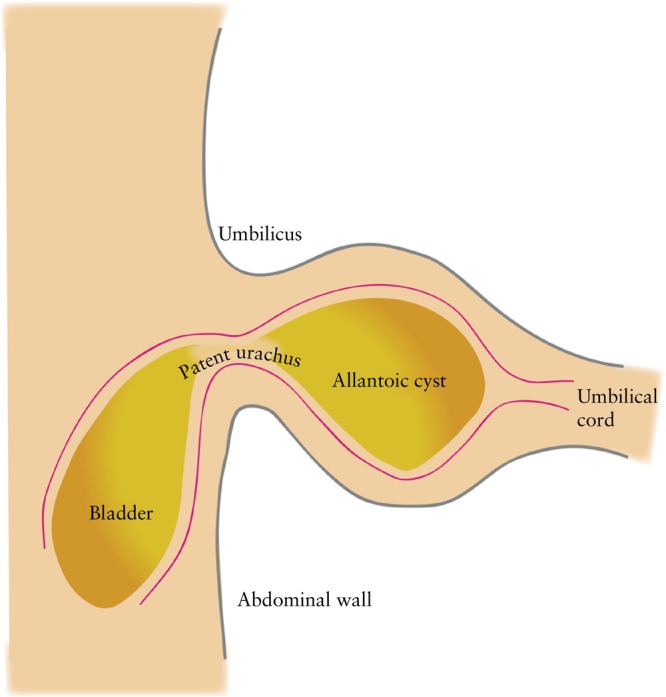
Representation of an allantoic cyst resulting from patent urachus.

In our cohort, early‐onset megacystis occurred in 77% of fetuses with megacystis associated with UCC, with a significant OR of 4.2, indicating an association between early‐onset fetal megacystis and UCC and suggesting a potential embryological origin^16^. The fetal urinary bladder develops from the urogenital sinus, continuous with the allantois, extending to the umbilical cord^17^. Normally, the allantois is obliterated between 6 and 12 weeks' gestation, leaving behind a structure, the urachus, which connects the umbilicus to the bladder apex (Figure 2)^18^, ^19^. Consequently, megacystis occurring before the obliteration of the allantois may result in a small drainage within the umbilical cord, hindering allantois closure and causing a patent urachus/allantoic cyst.

Furthermore, our study found significant clinical implications of UCC, including a 66% prevalence of complex diagnosis, primarily represented by ARM. Beyond fetal megacystis, UCC has been associated with chromosomal and structural defects, with a prevalence of 20% among all cases^18^. In our study, fetuses with UCC had 3.3 times higher odds of additional associated anomaly compared to fetuses without a pop‐off mechanism. Furthermore, the UCC group experienced the highest rate of IUFD observed in our study. UCC may predispose fetuses to umbilical cord hematoma near the fetal insertion, possibly due to vessel compression from the expanding cyst, leading to vessel wall ischemia and weakness as well as tearing of the umbilical cord^20^. Such hematomas could contribute to IUFD at any stage of pregnancy^20^.

### Extravasation

Extravasation of urine may result from elevated pressure within the urinary tract, leading to rupturing of the calyceal fornix and subsequent formation of ascites or urinoma^21^. This accounts for up to 30% of all cases of neonatal ascites^22^. Previous studies proposed antenatal ascites and urinoma as pop‐off mechanisms in fetuses with PUV, leading to improved renal function and lower rates of chronic kidney disease, and highlighted the clinical relevance of antenatal intervention^15^, ^21^, ^23^, ^24^. In our study, abnormal postnatal eGFR was more common in the group without pop‐off mechanisms, albeit not significantly so, suggesting that pop‐off mechanisms could exert a positive influence on postnatal eGFR. Among those with pop‐off mechanisms, abnormal eGFR was reported more frequently in the group with extravasation. This could be due to the inclusion of urinomas, which still exert pressure on the developing renal parenchyma, and the resorption capacity within the retroperitoneal space is less efficient than that in the peritoneum^25^.

The exact pathogenesis of extravasation remains unclear, but it is strongly linked to obstructions of the upper and lower urinary tract; more than 60% of urinomas are caused by LUTO^26^, ^27^, ^28^. This aligns with our findings, as LUTO emerged as the major underlying etiology of megacystis with extravasation.

### Megaureter/ureterocele

Increased intravesical pressure or obstruction can lead to ureteral dilatation in the fetal urinary system, as seen in conditions such as LUTO and vesicoureteral reflux. This dilatation could alleviate pressure within the bladder and facilitate urine flow. Our study found that fetuses with a megaureter/ureterocele exhibited the highest frequency of thickened bladder wall, which is likely due to the high prevalence of PUV associated with this condition and the presence of the keyhole sign^19^. Furthermore, fetuses with a megaureter/ureterocele had the highest likelihood of postnatal survival, which could be attributed to the low rate of additional associated anomaly and high rates of late‐onset megacystis and non‐severe megacystis. These fetuses also demonstrated the highest prevalence of normal eGFR postnatally, suggesting a potential protective effect of this pop‐off mechanism on kidney function, although this finding was not statistically significant.

### Strengths and limitations

One notable limitation of our study is the lack of routine examination of the entire umbilical cord, potentially resulting in an underestimation of the prevalence of UCC. Additionally, due to the retrospective design and lack of definitive postmortem or postnatal pathological confirmation, details about UCC type (i.e. allantoic cyst, pseudocyst or true cyst) were unavailable. Moreover, although there seemed to be a higher prevalence of normal eGFR among cases with an antenatal pop‐off mechanism, the lack of statistical significance may be attributed to the small number of available eGFR measurements in postnatally surviving patients. Additionally, fetuses without a pop‐off mechanism may have minimal or lower intravesical pressure compared to those with a pop‐off mechanism. To investigate more objectively the role of pop‐off mechanisms, antenatal intravesical pressure should be measured to provide more relevant insights^5^.

Strengths of this study include its large sample size, making it the first nationwide study on this topic across all academic centers in The Netherlands and providing a comprehensive representation of this condition. Additionally, our investigation highlights the role of UCC as a pop‐off mechanism, particularly in early‐onset fetal megacystis, which is a largely underexplored area.

### Conclusions

The presence of antenatal pop‐off mechanisms in fetuses with megacystis, occurring in 23% of cases, may crucially alleviate intraluminal pressure. UCC is associated with early‐onset and severe megacystis, normal AFI and high prevalence of complex diagnosis, notably ARM. Extravasation is associated with severe megacystis, abnormal AFI, the keyhole sign and additional associated anomaly. Megaureter/ureterocele is associated with late‐onset megacystis, a thickened bladder wall and low prevalence of additional associated anomaly. Fetuses presenting with extravasation had the highest rates of termination of pregnancy and non‐survivors, while those with UCC had the highest rate of IUFD, compared to the other groups. In contrast, fetuses with a megaureter/ureterocele demonstrate the most favorable outcomes regarding survival. Understanding the manifestations and outcomes associated with the three described pop‐off mechanisms in the context of fetal megacystis could inform antenatal management strategies and parental counseling.

## Data Availability

Data is available upon request.
